# Supplementary material for: Suboptimal MMR Vaccination Coverages—A Constant Challenge for Measles Elimination in Romania
Source: Vaccines (Basel). 2024 Jan 22;12(1):107. doi: 10.3390/vaccines12010107 (PMC10819452; doi:10.3390/vaccines12010107)
Supplement: Supplementary file 1 [file vaccines-12-00107-s001.zip › vaccines-2769492-supplementary.pdf]

Anex A

***Table S1. Estimation Activity Results Reporting Form a vaccination coverage at the age of 12 months,
held in August 2023 (children born in July 2022)***

County Public Health Directorate/Regional Centre of Public Health ________________

Estimation of vaccination coverage (1)												
Vaccine type	Number of children with the indicated number of vaccine doses in the vaccination history											
	Urban						***Rural***					
	4	3	2	1	0	***VC*** (%)	4	3	2	1	0	***VC*** (%)
BCG (Calmette – Guerin tuberculosis)												
Hepatitis B paediatric												
DTPa (diphthaeria, tetanus, pertussis acellular)												
Hib (Haemophilus influenzae b)												
VPI (poliomyelitis)												
Pneumococcal												
MMR (measles, mumps, rubella)												
Total number of children investigated for vaccination antecedents (history)	***Urban =***						***Rural =***					

Evaluation of age-appropriate reasons for non-vaccination (2)				
Reasons for non-vaccination	Number of unvaccinated children according to age, due to the reasons mentioned below			
Medical contraindication (C)	**Contraindication type**	**Number of unvaccinated children**	**Contraindication type**	**Number of unvaccinated children**
• If yes, mention the numbers not vaccinated with temporary or definitive C	temporary		temporary	
	final		final	
• Specify which definitive C and<br>• no. unvaccinated against type C				
Parent/belonging refusal (R)	**Refusal type/ vaccine**	**Number of unvaccinated children**	**Refusal type/ vaccine**	**Number of unvaccinated children**
• If yes, general refusal (for all vaccines) or refusal for a certain type of vaccine	general		general	
	BCG (Calmette – Guerin tuberculosis)		BCG (Calmette – Guerin tuberculosis)	
	Hep B		Hep B	
	DTPa		DTPa	
	Hib		Hib	
	VPI		VPI	
	Pneumococcal		Pneumococcal	
	MMR		MMR	
No show/No announcement (N)				
Born/left abroad (S)				
Omission (O)				
Lack of vaccine (L)	**Vaccine type**	**Number of unvaccinated children**	**Vaccine type**	**Number of unvaccinated children**
• If there is no vaccine, yes, mention the number of unvaccinated for each type of vaccine	BCG		BCG	
	Hep B		Hep B	
	DTPa		DTPa	
	Hib		Hib	
	VPI		VPI	
	Pneumococcal		Pneumococcal	
	MMR		MMR	

**_Total number of unvaccinated children according to age_**	**_Urban =_**	**_Rural =_**

Number of vaccinating family doctors _________
National Electronic Vaccination Registry (NEVR) Survey:

- No. of family doctors verified by survey (**minimum 30% of the number of vaccinating doctors**) = ________
- No. of family doctors where the results of the estimation of vaccination coverage, carried out according to the methodology, correspond to those in the NEVR = _________

Date : _ _ / _ _ / _ _ _ _ **Epidemiologist MD** ______________

Legend for Anex A:

(1) Source of information: Child's medical consultation sheet, The register of vaccinations-revaccinations, tabel with monthly number of eligible children for vaccination, the National Electronic Register of Vaccinations

(2) Source of information: Child's medical consultation sheet

(C) = child with recorded visits to the family doctor's office and with at least one medical contraindication recorded in the child's medical consultation card, signed and initialed by the family doctor

(R) = child whose parent/belonging refuses to vaccinate the child and who certifies the refusal by signing the registration table; for parents who refuse to sign, the family doctor will mention this, under the signature, and the epidemiologist will check in the field, by survey, if this aspect is confirmed

(N) = child without recorded visits to the medical office

(S) = child born abroad, not vaccinated according to the age or whose parent/relative cannot prove, with medical documents, the vaccination according to the age

(O) = eligible child who has not been registered for vaccination

(L) = eligible child who has not been vaccinated due to lack of vaccine

(AV) = vaccination coverage = number of children vaccinated according to age X 100/total number of children investigated for vaccination antecedents
